# Response of Basil Growth and Morphology to Light Intensity and Spectrum in a Vertical Farm

**DOI:** 10.3389/fpls.2020.597906

**Published:** 2020-12-04

**Authors:** Dorthe H. Larsen, Ernst J. Woltering, Celine C. S. Nicole, Leo F. M. Marcelis

**Affiliations:** ^1^Horticulture and Product Physiology, Department of Plant Sciences, Wageningen University & Research, Wageningen, Netherlands; ^2^Postharvest Technology, Wageningen Food and Biobased Research, Wageningen University & Research, Wageningen, Netherlands; ^3^Signify, Eindhoven, Netherlands

**Keywords:** basil, LED, spectra, blue light, far-red light, photosynthetic photon flux density, vertical farming

## Abstract

Vertical farming is becoming increasingly popular for production of leafy vegetables and herbs, with basil (*Ocimum basilicum* L.) as one of the most popular herbs. In basil most research has focused on increasing secondary metabolites with light spectra. However, knowledge about the effect of light intensity (photosynthetic photon flux density, PPFD) and spectra on growth and morphology is key for optimizing quality at harvest. The impact of PPFD and spectrum on plant growth and development is species dependent and currently few studies in basil are available. Understanding the response to End-Of-Production (EOP) light of growth and morphology is important for successful vertical farming. We performed a comprehensive series of experiments, where the effects of EOP PPFD, fraction of blue and their interaction on the growth and morphology were analyzed in two green and one purple basil cultivar. In addition, the impact of different EOP intensities and duration of far-red were investigated. We found that increasing the PPFD increased fresh mass, dry matter content and plant height in all three cultivars. The responses were linear or quadratic depending on the cultivar. A high fraction of blue (>90%) increased plant height and decreased the dry mass partitioning to the leaves. The only interaction found between the fraction of blue and overall PPFD was on plant height in the green cultivar whereas other growth parameters and morphology responded stronger to PPFD than to the fraction of blue light. Plant dry matter production was increased with the addition of far-red. Far-red EOP intensity treatments enhanced the fraction of dry mass partitioned to the leaves, whereas a prolonged far-red treatment enhanced partitioning to the stem. Both plant fresh mass and dry matter content were improved by applying high PPFD shortly before harvest. Light spectra were found to be of less importance than PPFD with respect to plant dry matter content. Light use efficiency (LUE) based on fresh mass decreased with increasing PPFD whereas LUE based on dry mass increased with increasing PPFD, when given as EOP treatments. The overall physiological mechanisms of the light intensity and spectral effects are discussed.

## Introduction

Vertical farming systems, also called plant factories, are indoor growth facilities with plants grown in multiple layers. In a vertical farm, plants are grown in a closed system without the use of pesticides and all climate factors can be controlled (SharathKumar et al., 2020). Controlling the pre-harvest factors can have a great influence on the growth and morphology as well as postharvest quality (Mattheis and Fellman, 1999). Light is the primary source of energy for plants and the dominant light source in a vertical farm is light emitting diodes (LEDs) which makes a vertical farm efficient and allows for year-round production. LEDs are energy efficient, they have a low heat emission, the light intensity can be adjusted and light spectra can be modulated (Kusuma et al., 2020). Leafy vegetables and herbs are often the crops of choice in vertical farms due to fast growth, low plant height, and high retail price (Touliatos et al., 2016). One popular culinary herb is basil (*Ocimum basilicum* L.) that is used for its unique aroma. Besides aroma other important quality parameters include yield, plant morphology and fresh mass and dry matter content (Maness, 2003; Zhou et al., 2012). However, there has been little research on elucidating the response to light intensity and spectra of yield and dry matter content in basil.

Plant development, yield and dry matter content are highly affected by light intensity. Light intensity used for photosynthesis is defined as photosynthetic photon flux density (PPFD) ranging from 400–700 nm (McCree, 1972; Poorter et al., 2019). Increased light intensity generally correlates with an increase in net photosynthesis which can increase plant fresh mass and yield. Furthermore, an increase in light intensity can increase soluble sugars which are part of the dry matter. In basil, plant growth and dry matter content were found to increase under increasing light intensity but only until an optimum after which the plants might be limited by other environmental factors (Pennisi et al., 2020). Yet, Kelly et al. (2020) found in lettuce biomass increased linearly with PPFD. In addition to PPFD, light spectrum is important for morphological features, specifically the partitioning of carbon to leaves vs. stem. Some of the most studied light spectra include ratios of blue (400–500 nm) and red (600–700 nm), and addition of far-red (700–800 nm) to PPFD. While red is the most efficient color for photosynthesis and energy use, 100% red often disturbs normal morphology (i.e., leaf curling, thin, and pale leaves). It is important to add blue to the spectra for optimal morphology. Blue light plays a role in several plant processes such as photomorphogenesis, stomatal opening, and leaf photosynthetic functioning (Hogewoning et al., 2010; Boccalandro et al., 2012). An optimum of blue light could exist for photosynthetic capacity as well as for biomass accumulation (Kaiser et al., 2019). Fresh mass (Li and Kubota, 2009) and dry matter (Kalaitzoglou et al., 2019) can also increase with the addition of far-red. Furthermore, far-red has been associated with increased leaf area and plant height in basil (Carvalho et al., 2016) which could increase light interception. Plant height might also increase under 100% blue light (Heo et al., 2002; Johnson et al., 2020). However, the opposite effect has been reported in several studies where a high fraction of blue light resulted in more compact plants (Hoenecke et al., 1992; Islam et al., 2012; Keuskamp et al., 2012). In basil, contradictory reports exists with respect to the plant growth and morphology response to light spectra. Plant height was neither affected by 100% blue light compared to greenhouse grown basil (Carvalho et al., 2016), nor the addition of blue to a red light spectra (20–60% blue) in a greenhouse (Jensen et al., 2018), or the response to blue light was entangled with addition of far-red (Bantis et al., 2016). Piovene et al. (2015) reported 37% blue had a positive effect while Pennisi et al. (2019) found that a fraction of blue above 30% had a negative effect on fresh mass. However, the optimal PPFD, as well as spectra with respect to fraction of blue and addition of far-red light for plant growth have been found to be highly species dependent (Kim et al., 2006; Colonna et al., 2016).

Several studies have focused on increasing the secondary metabolites in basil, however, fewer studies have elucidated the effect of PPFD, light spectra and the interaction of the two on the growth and morphological features. The primary attribute of crops in a commercial production system is biomass, that is fresh and dry mass of leaves. Other relevant attributes include morphology such as short internodes and increased partitioning of carbon to the leaves. Knowledge of the response of basil to changes in light intensity and spectra will allow for a fully controlled plant production and a desired growth and morphology. To optimize production in vertical farming, it has been proposed to focus the lighting strategy during the first part of cultivation cycle on optimizing biomass increase, while the last period before harvest the lighting strategy should focus on optimizing product quality by End-Of-Production (EOP) treatments (SharathKumar et al., 2020). We aimed at understanding the response of growth and morphology of basil to PPFD, fraction of blue light and far-red. In addition, we wanted to study the response to EOP light applied 5–7 days before harvest. To study this, we set up a comprehensive series of (five) studies, in a vertical farming set-up with green and purple basil cultivars.

## Materials and Methods

### Growth Conditions

Basil (*O. basilicum* L.) was grown in a climate chamber in a vertical farming set-up with twelve compartments of the size 0.8 × 1.3 m in table area and a plant density of 123 plants m^–2^. Two green cultivars (Emily and Dolly) and one purple cultivar (Rosie) Sweet basil, were used; all cultivars were derived from Enza Zaden, NL. Seeds were germinated under red-white LED light (GreenPower LED production module 120 cm, DeepRedWhite, Phillips Eindhoven, Netherlands) varying between 150 and 200 μmol m^–2^ s^–1^ (Table 1). The spectral intensities in Experiment 1–4 were measured with a spectroradiometer (USB2000 spectrometer, Ocean Optics, Duiven, Netherlands), and in Experiment 5 with another spectroradiometer (SS-110; Apogee Instruments, Logan, UT, United States). Phytochrome Photostationary state (PSS) values were calculated according to Sager et al. (1988). PPFD was regularly measured with a quantum sensor (LI-190SB quantum sensor, LI-1400 Datalogger, LI-COR Bioscience, Lincoln, NE, United States) to adjust the height of the light frames during the growth and maintain a constant light intensity at the top of the plants throughout each experiment. The sides of each compartment were covered with white reflective plastic to increase light uniformity. Seeds were sown in trays with 240 stone wool plugs (Grodan Rockwool B.V., Netherlands) with one seed per plug. After 10–15 days the morphologically most similar plants were selected and transplanted to 7.5 × 7.5 × 6.5 cm stone wool blocks (Grodan Rockwool B.V., Netherlands), one outer row surrounding the plants were border plants and not used for the experiment. Day/night temperature was kept at 25°C, the relative humidity was set at 75% and CO_2_ was ambient concentration. Relative humidity and temperature in each light treatment were recorded with either keytag dataloggers (KTL-508, Keytag, NL) or Hanwell data loggers (ML4160, Hanwell Solutions, United Kingdom) with deviations within 10% and 1°C from the set points. To maintain air temperature around 25°C fans were installed in high light treatments above the lamps blowing out of the individual compartments. Plants were kept well-watered through an ebb and flood system based on plant needs and growth stage. For the first 3 weeks of the growth, plants were watered once every second day for 10 min and after that once every day for 10 min. High light and high blue treatments were given an extra round of watering when needed. The nutrient solution consisted of NO_3_^–^ 8.5 mM, SO_4_^2–^ 1.5 mM, HPO_4_^2–^ 1.5 mM, NH_4_^+^ 1.5 mM, K^+^ 5.5 mM, Ca^2+^ 4.0 mM, Mg^2+^ 1.5 mM, Cl^–^ 0.2 mM, Fe^3+^/Fe^2+^ 30 μM, Mn^2+^ 5 μM, Zn^2+^ 5 μM, H_2_BO_3_^–^ 35 μM, Cu^+^/Cu^2+^ 1 μM, MoO_4_^2–^ 1 μM with pH 5.7 and EC 1.7 dS m^–1^ before transplant and with an EC of 2.3 dS m^–1^ after transplant.

**TABLE 1:**
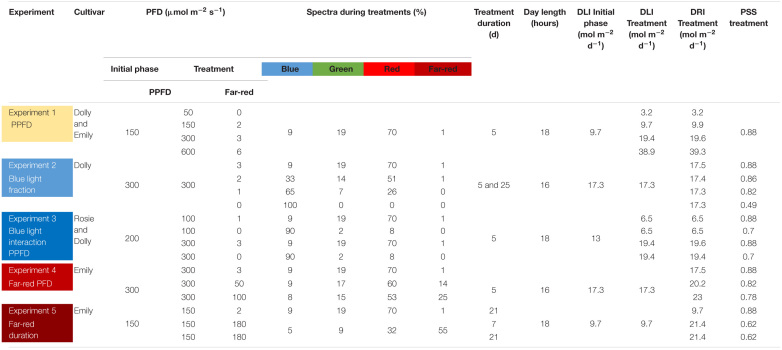
Overview of the experiments carried out.

*PPFD (400–700 nm) and PFD of far-red (700–800 nm) during the initial phase (i.e., from transplant until start of treatments) and treatments, and spectral composition during the treatments. Fractions of the spectra; blue (400–500 nm), green (500–600 nm), red (600–700 nm), and far-red (700–800 nm) are percentages of the total Photon flux density (PFD) (400–800 nm), treatment duration, the daily light integral (DLI) from 400–700 nm for the initial phase, DLI and daily radiation integral (400–800 nm) and treatments are given. Phytochrome photostationary state (PSS) were calculated according to Sager et al. (1988).*

### Experimental Set-up

Five different experiments were performed (summarized in Table 1). In Experiment 1 the response of cultivars Emily and Dolly to different light intensities applied as EOP treatments during 5 days before harvest was investigated. Seeds of both cultivars germinated for 15 days under 150 μmol m^–2^ s^–1^. After transplant, the light intensity was kept at 150 μmol m^–2^ s^–1^ for another 15 days. During the whole growth period a light spectrum with red-white LED was used and a day length of 18 h. EOP treatments were given for 5 days and included light intensities of 50, 150, 300, and 600 μmol m^–2^ s^–1^, respectively.

In Experiment 2 the response of cultivar Dolly to different fractions of blue light applied either throughout the growth (25 days) or as EOP treatment during 5 days before harvest were investigated. The different fractions of blue light were created by using different ratios between pure blue (GreenPower LED production module, 120 cm, Blue, Phillips Eindhoven, Netherlands) and red white LEDs. Seeds germinated for 15 days under 150 μmol m^–2^ s^–1^ red white LED light. After transplant the plants were exposed for 25 days to four different blue light (400–500 nm) treatments of 9, 33, 65, and 100% out of the total PPFD of 300 μmol m^–2^ s^–1^. In three other treatments the plants were grown under red white light of 300 μmol m^–2^ s^–1^ for 20 days after which they were exposed for 5 days to different blue light treatments of 33, 65, and 100%. Day length was 16 h.

In Experiment 3 the response of cultivars Rosie and Dolly to EOP treatments with increased fractions of blue light and the interaction with PPFD during 5 days before harvest were investigated. Seeds of both cultivars germinated under 200 μmol m^–2^ s^–1^ red white LED light for 15 days. After transplant the plants were grown for another 15 days under red white light of 200 μmol m^–2^ s^–1^. Five days before harvest plants were exposed to treatments of 100 μmol m^–2^ s^–1^ PPFD with 9% blue, 100 μmol m^–2^ s^–1^ PPFD with 90% blue, 300 μmol m^–2^ s^–1^ PPFD with 9% blue and 300 μmol m^–2^ s^–1^ PPFD with 90% blue. The different fractions blue light were created by using different ratios between pure blue (GreenPower LED production module, 120 cm, Blue, Phillips Eindhoven, Netherlands) and red white LEDs. Day length was 18 h.

In Experiment 4 the response of cultivar Emily to EOP treatments with increasing intensities of far-red in addition to the PPFD during 5 days before harvest were investigated. Seeds germinated under 150 μmol m^–2^ s^–1^ red white LED light for 15 days. After transplant the PPFD was increased to 300 μmol m^–2^ s^–1^ of red white LED light for 15 days. EOP treatments were applied 5 days before harvest with 0, 50, or 100 μmol m^–2^ s^–1^ far-red (GreenPower Production module, 120 cm, Far Red, Phillips Eindhoven, Netherlands) added to the 300 μmol m^–2^ s^–1^ of red white LED light. This resulted in treatments with a total photon flux density (PFD) of 303, 350, 400 μmol m^–2^ s^–1^ (400–800 nm). Day length was 16 h.

In Experiment 5 the response of cultivar Emily to different durations of far-red before harvest were investigated. Seeds germinated under 150 μmol m^–2^ s^–1^ red white LED light for 10 days. After transplant the plants continued to grow under 150 μmol m^–2^ s^–1^ red white light for another 21 days. No far-red was applied or additional far-red (GreenPower Production module, 120 cm, Far Red, Phillips Eindhoven, Netherlands) (180 μmol m^–2^ s^–1^) was applied during 1 week (as EOP treatment) or 3 weeks (throughout the growth). This resulted in treatments with a total PFD of 152, 330, 330 μmol m^–2^ s^–1^ (400–800 nm). Day length was 18 h.

### Measurements of Growth and Morphological Parameters

Plant height was measured from the surface of the stone wool block to the height of the apex. Leaves of a minimum size of 1 cm^2^ were counted as true leaves, leaf area was measured with a leaf area meter LI-3100C (LICOR, Lincoln, NE, United States). Leaves and stem were separated and weighed for fresh mass and dry mass. Dry mass was measured after drying for 48 h at 80°C.

Daily light integral (mol_400–700 *nm*_ m^–2^ d^–1^) was calculated as:

(1) $$\text{PPFD}\left(\mu \;\mathrm{mol}\;{\text{m}}^{-2}{\text{s}}^{-1}\right)\times \mathrm{day}\mathrm{length}\left(\text{h}\right)\times 0.0036$$

Daily radiation integral (mol_400–800 *nm*_ m^–2^ d^–1^) was calculated as:

(2) $$\text{PFD}\left(\mu \;\mathrm{mol}\;{\text{m}}^{-2}{\text{s}}^{-1}\right)\times \mathrm{day}\mathrm{length}\left(\text{h}\right)\times 0.0036$$

Light use efficiency (g mol_400–700 *nm*_^–1^) was calculated as:

(3) $$\begin{array}{c} \frac{\mathrm{plant}\mathrm{mass}\left(\text{g}\right)\times \mathrm{plant}\mathrm{density}\left(\mathrm{plants}{\text{m}}^{-2}\right)}{\mathrm{Daily}\mathrm{Light}{\mathrm{Integral}}_{\left(400-700\mathrm{nm}\right)}\left(\mathrm{mol}{\text{m}}^{-2}{\text{d}}^{-1}\right)}\\ \times \mathrm{days}\mathrm{of}\mathrm{cultivation}\left(d\right) \end{array}$$

Radiation use efficiency (g mol_400–800 *nm*_^–1^) was calculated as:

(4) $$\begin{array}{c} \frac{\mathrm{plant}\mathrm{mass}\left(\text{g}\right)\times \mathrm{plant}\mathrm{density}\left(\mathrm{plants}{\text{m}}^{-2}\right)}{\mathrm{Daily}\mathrm{Radiation}{\mathrm{Integral}}_{\left(400-800\mathrm{nm}\right)}\left(\mathrm{mol}{\text{m}}^{-2}{\text{d}}^{-1}\right)}\\ \times \mathrm{days}\mathrm{of}\mathrm{cultivation}\left(d\right) \end{array}$$

Specific leaf area (SLA) (cm^2^ g^–1^) was calculated as:

(5) $$\frac{\mathrm{Leaf}\mathrm{area}\left({\mathrm{cm}}^{2}\right)}{\mathrm{leaf}\mathrm{dry}\mathrm{mass}\left(g\right)}$$

Dry matter content (%) was calculated as:

(6) $$\frac{\mathrm{dry}\mathrm{mass}\left(g\right)}{\mathrm{fresh}\mathrm{mass}\left(g\right)}\times 100\%$$

### Statistical Set-up and Analysis

The experiments were carried out as complete randomized block designs. Each experiment was repeated in time, which represented the blocks. In each experiment six small compartments were used for plant growth. Each light treatment was done in one compartment and repeated in time. For each repetition the position of the light treatments in the six compartments were randomized. Generally 5 or 6 representative plants from the light compartment were sampled for the analyses. For statistical analyses, the average values of each block were used as one replicate. Experiment 1 and 3 (cv. Dolly) were carried out three times, experiment 3 (cv. Rosie) 4 times and experiment 2, 4, and 5 and two times.

Data was analyzed with Genstat (VSN International, 19th Edition). Experiments on light intensity, blue light and far-red were analyzed with One-way Analysis of Variance (ANOVA), while the blue and blue light × light intensity experiment were analyzed with a two-way ANOVA, followed by *post hoc* LSD test. Treatment effects were tested at a probability level of 5%, unless an experiment had only two blocks in which case probability level of 10% was applied (Ott and Longnecker, 2010). Furthermore, it was tested with the ANOVA if a polynomial model could explain the effect of the light treatment on the tested variates. Significance of the linear or quadratic component were used as proof of treatment having a significant effect (and additionally if this effect was linear or quadratic). Based on the result of the ANOVA a linear or quadratic trendline was added in Excel (Excel, Microsoft Pro Plus 2019). When no interaction was found in the two-way ANOVAs the overall means were shown. Assumptions of homogeneity and normality were met as tested with Bartlett’s and Shapiro–Wilk test, respectively.

## Results

### Response to End-Of-Production PPFD (Experiment 1)

**FIGURE 1:**
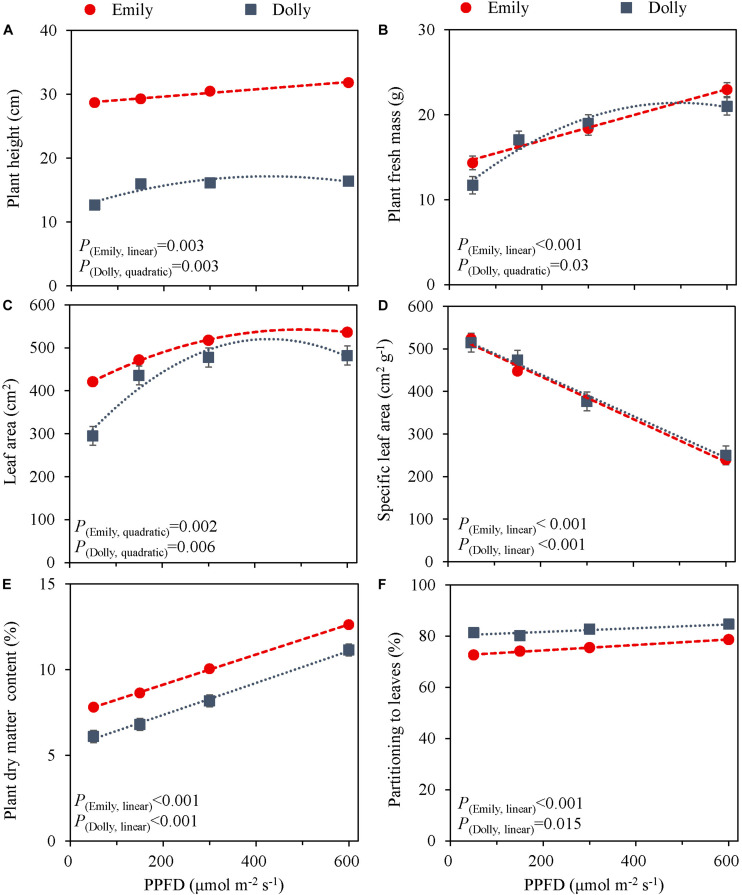
Response of basil cvs. Emily (red circles) and Dolly (gray squares) to different End-Of-Production PPFD (Experiment 1). Plants were grown for 30 days under 150 μmol m^–2^ s^–1^ after which they were exposed to different PPFD (i.e., 50, 150, 300, and 600 μmol m^–2^ s^–1^) during 5 days before harvest. **(A)** Plant height, **(B)** plant fresh mass, **(C)** leaf area, **(D)** specific leaf area, **(E)** plant dry matter content, **(F)** dry mass partitioning to leaves. Data are means of three blocks (*n* = 3) each with six replicate plants. Error bars represent standard errors of means, when larger than symbols. For significant quadratic or linear effects of PPFD, trendlines together with the respective *p*-values (α = 0.05) are depicted.

The PPFD during the last 5 days had a significant effect on all growth parameters in cultivars Emily and Dolly. Plant height, plant fresh mass, leaf area and partitioning of dry mass to the leaves all increased with an increase in PPFD (Figure 1). The response to PPFD was linear or quadratic depending on the different parameters and cultivars. Plant fresh mass displayed a significant linear response to light intensity for cv. Emily while it was a quadratic response for cv. Dolly, indicating that within this range an optimum PPFD exists for cv. Dolly (Figure 1B). A similar trend was found for plant height between the two cultivars (Figure 1A), whereas both cultivars had a quadratic response to light for leaf area (Figure 1C). Plant dry matter content and partitioning to the leaves increased linearly with increase in PPFD for both cultivars (Figures 1E,F). SLA decreased due to a strong increase in dry mass of the leaves for both cultivars (Figure 1D). Plants from both cultivars grown under 600 μmol m^–2^ s^–1^ displayed very brittle leaves that easily broke at the petiole and broke easily when handled. For both cultivars light use efficiency (LUE) based on dry mass increased with increasing PPFD, but decreased when based on fresh mass (Table 2).

**TABLE 2:**
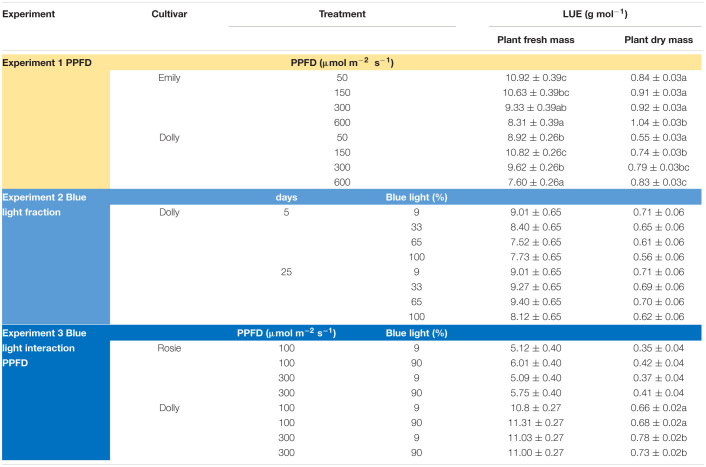
Overview of light use efficiency (LUE) for plant fresh and dry mass in response to PPFD and fraction blue (Experiment 1–3).

*LUE is based on PPFD incident on the plants accumulated over the initial (i.e., from transplant until start of treatments) and treatment phase. Letters indicate significant differences.*

### Response to Increasing Fraction Blue Light in the Light Spectrum (Experiment 2)

**FIGURE 2:**
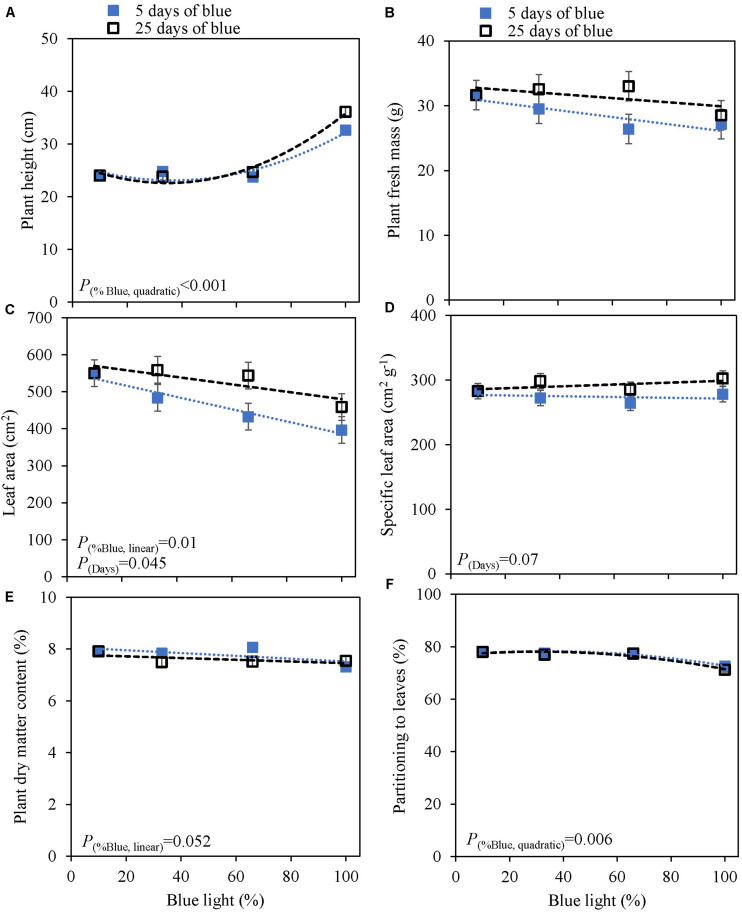
Response of basil cv. Dolly to different blue fractions out of a total PPFD of 300 μmol m^–2^ s^–1^ either applied throughout the growth for 25 days (open squares) or as 5 days End-Of-Production treatments (closed squares) (Experiment 2). The data point 9% blue is shared between 5 and 25 days as 9% blue light also was the initial phase before EOP treatments. **(A)** Plant height, **(B)** plant fresh mass, **(C)** leaf area, **(D)** specific leaf area, **(E)** plant dry matter content, **(F)** dry mass partitioning to leaves. Data are means of two blocks (*n* = 2) each with six replicate plants. Error bars representing standard errors, when larger than symbol size. For significant quadratic or linear effects of increasing fraction of blue, trendlines together with the respective *p*-values (α = 0.10) are depicted.

The response to varying fractions of blue light was studied in a 5 day EOP treatment and as a throughout the growth treatment (25 days) in cv. Dolly. Plants showed a fairly similar response to the fraction of blue light, both to the EOP and throughout the growth treatments. The largest difference was found between the 100% blue treatment and the other treatments which also included red and green light. Plant height increased quadratically while the fraction of dry mass partitioned into the leaves decreased quadratically with increasing blue light (Figure 2); in fact it only showed a strong response when the fraction of blue was raised to 100%. The leaf area (Figure 2C), leaf fresh and leaf dry mass (Supplementary Figures 2A,C) decreased linearly with increasing fraction of blue light. There was no appreciable effect on the dry matter content of the leaves. LUE based on both dry and fresh mass did not significantly change with neither fraction of blue light or number of treatment days (Table 2). The only difference found between 5 and 25 days of application of blue light was on leaf area with increases in SLA when grown under 25 days of increased fraction of blue light.

### Interaction Between Fraction of Blue Light and PPFD (Experiment 3)

**FIGURE 3:**
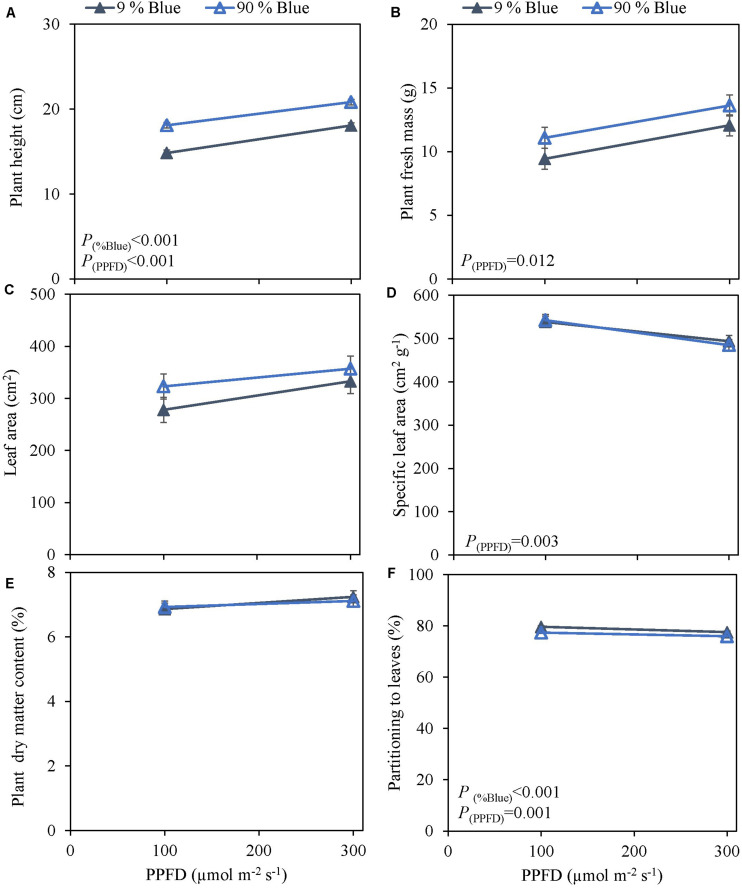
Response of basil cv. Rosie to End-Of-Production blue light and PPFD. Plants were grown for 30 days under red white light (9% blue) and PPFD of 200 μmol m^–2^ s^–1^ (Experiment 3). EOP treatments were applied 5 days before harvest blue light and PPFD were changed to 100 μmol m^–2^ s^–1^ red white with 9% blue or 90% blue, and to 300 μmol m^–2^ s^–1^ red white with 9% blue or 90% blue. Closed triangle 9% blue and open triangle 90% blue. **(A)** Plant height, **(B)** plant fresh mass, **(C)** leaf area, **(D)** specific leaf area, **(E)** plant dry matter content, **(F)** dry mass partitioning to leaves. Data are means of four blocks (*n* = 4) each with six replicate plants. Error bars representing standard errors, when larger than symbol size. *p*-Values of main effects % Blue and PPFD (α = 0.05) are depicted.

**FIGURE 4:**
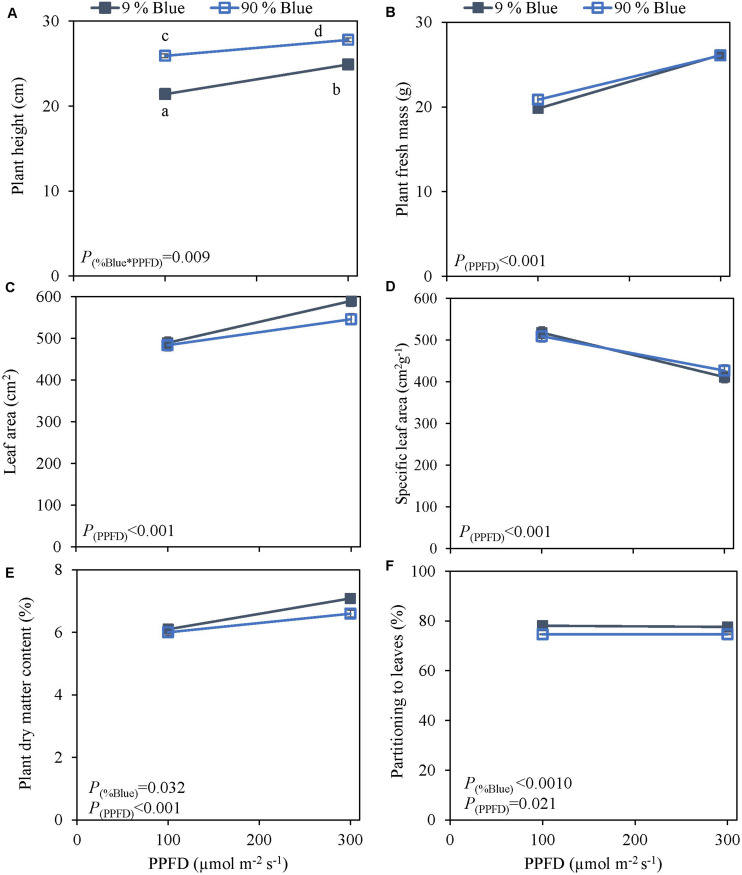
Response of basil cv. Dolly to End-Of-Production blue light and PPFD. Plants were grown for 30 days under red white light (9% blue) and PPFD of 200 μmol m^–2^ s^–1^ (Experiment 3). EOP treatments were applied 5 days before harvest blue light and PPFD were changed to 100 μmol m^–2^ s^–1^ red white with 9% blue or 90% blue, and to 300 μmol m^–2^ s^–1^ red white with 9% blue or 90% blue. Closed squares 9% blue and open squares 90% blue. **(A)** Plant height, **(B)** plant fresh mass, **(C)** leaf area, **(D)** specific leaf area, **(E)** plant dry matter content, **(F)** dry mass partitioning to leaves. Data are means of four blocks (*n* = 3) each with six replicate plants. Error bars representing standard errors, when larger than symbol size. *p*-Values of main effects %Blue and PPFD (α = 0.05) are depicted.

Photosynthetic photon flux density could play an important role in the response to blue light. Therefore the interaction of PPFD and fraction of blue light was studied in a purple (Rosie) and a green cultivar (Dolly) (Figures 3, 4). The green leaved cultivar (Dolly) showed only a limited response to blue light in Experiment 2, therefore we here extended the experiment with a cultivar with purple leaves. In this way we could test if the response to the light depended on the color (i.e., content of anthocyanins) of the leaves. Plant height (Figures 3A, 4A) was higher at high PPFD compared to low PPFD (100 vs. 300 μmol m^–2^ s^–1^) and height was higher at 90% compared to 9% blue light for both cultivars. These results were similar as in the experiments where either the PPFD or the fraction blue were studied separately (Figures 1, 2). For cv. Dolly the response of plant height to blue light was greater at a lower PPFD (about 20% increase) than at higher PPFD (10%) whereas for the purple cultivar Rosie no interaction between blue light and PPFD was found. The increase in plant height corresponded to an increase in fresh mass of stems (Supplementary Figures 3B, 4B) and a lower dry mass partitioning to the leaves with higher light intensity and fraction of blue (Figures 3F, 4F). Moreover, cv. Rosie responded mainly to the increase in PPFD while cv. Dolly had an increase in plant dry matter content and dry mass of leaves with an increase in both fraction of blue and PPFD. The LUE based on plant dry mass increased for cv. Dolly when PPFD was increased from 100 to 300 μmol m^–2^ s^–1^whereas no change in LUE was found for cv. Rosie (Table 2).

### Response to Increasing Far-Red Intensities and Duration (Experiment 4 and 5)

**FIGURE 5:**
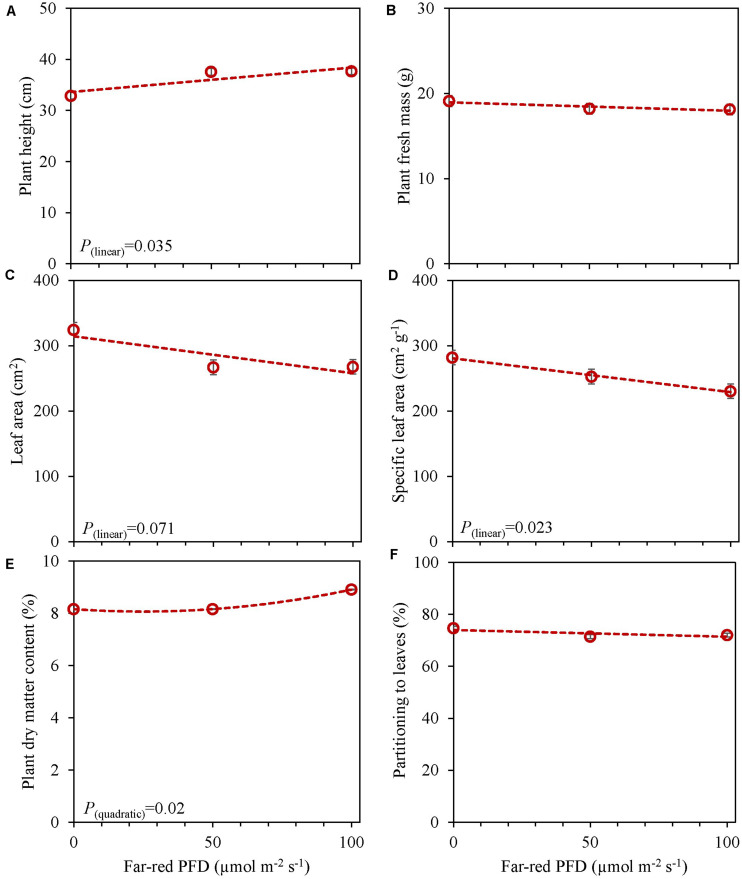
Response of basil cv. Emily to End-Of-Production increased far-red PFD (Experiment 4). Plants were grown for 15 days under PPFD 150 μmol m^–2^ s^–1^, after transplant for another 15 days of PPFD 300 μmol m^–2^ s^–1^ red white light and exposed to different far-red intensities (i.e., 0, 50, 100 μmol m^–2^ s^–1^) in addition to 300 μmol m^–2^ s^–1^ red white light applied during 5 days before harvest. **(A)** Plant height, **(B)** plant fresh mass, **(C)** leaf area, **(D)** specific leaf area, **(E)** plant dry matter content, **(F)** dry mass partitioned to leaves. Data are means of two blocks (*n* = 2) each with five replicate plants. Error bars representing standard errors, when larger than symbol size. For significant quadratic or linear effects of increasing far-red intensity, trendlines together with the respective *p*-values (α = 0.10) are depicted.

**FIGURE 6:**
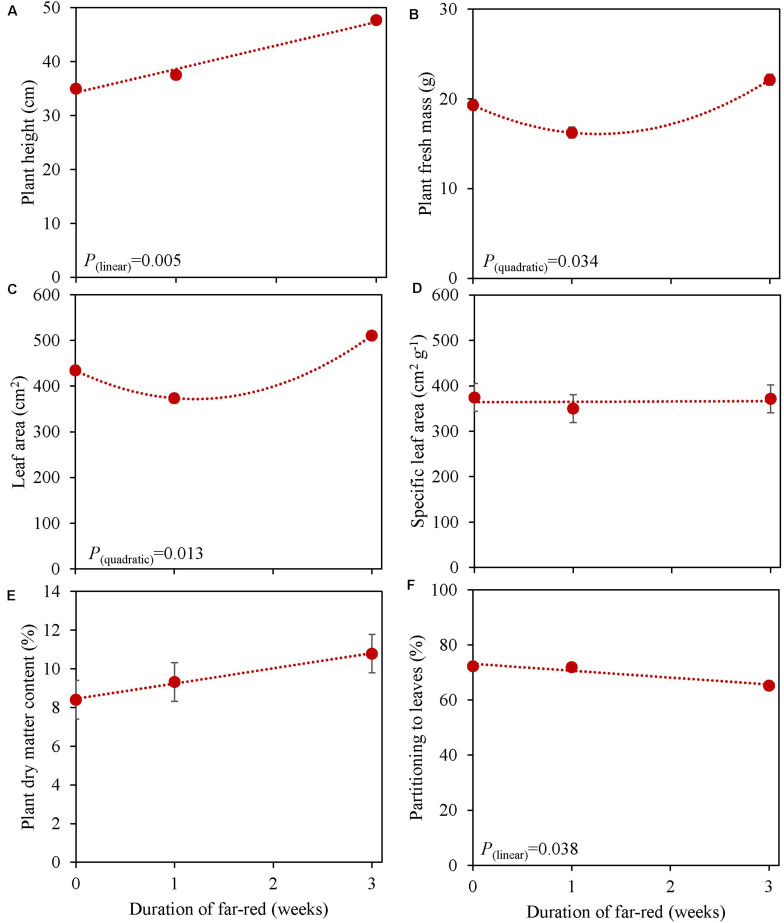
Response of basil cv. Emily to different duration of far-red treatments either throughout the growth for 3 weeks or as 1 week End-Of-Production treatment (Experiment 5). Plants were grown for 31 days under 150 μmol m^–2^ s^–1^ red white light, and additional far-red light (180 μmol m^–2^ s^–1^) was applied during 0, 1 and 3 weeks before harvest. **(A)** Plant height, **(B)** plant fresh mass, **(C)** leaf area, **(D)** specific leaf area, **(E)** plant dry matter content **(F)** dry mass partitioning to leaves. Data are means of two blocks (*n* = 2) each with five replicate plants. Error bars representing standard errors, when larger than symbol size. For significant quadratic or linear effects of duration of far-red, trendlines together with the respective *p*-values (α = 0.10) are depicted.

In an experiment with cv. Emily, 5 days EOP treatments were applied adding 0, 50, or 100 μmol m^–2^ s^–1^ far-red to the PPFD of 300 μmol m^–2^ s^–1^ red-white light (Figure 5). In another experiment different durations of (180 μmol m^–2^ s^–1^) far-red were applied for 0, 1, and 3 weeks (Figure 6) on top of 150 μmol m^–2^ s^–1^ red-white light. Plant height increased by 15% with the addition of 100 μmol m^–2^ s^–1^ of far-red and 7 and 36% with the one and 3 weeks duration of far-red, respectively. The increase in plant height was significant as the linear component of the statistical analysis was significant. Other responses to duration and intensity of far-red in terms of fresh, dry mass, dry matter content and SLA differed greatly. Increased intensity of EOP far-red on top of 300 μmol m^–2^ s^–1^ red-white light resulted in a significant decrease in leaf area and s SLA while a small increase in plant dry matter content was observed. Plant fresh mass and partitioning to leaves did not respond to increased intensity of far-red when given on top of 300 μmol m^–2^ s^–1^ red-white light during 5 days. Interestingly plant dry matter content increased with the addition of 100 μmol m^–2^ s^–1^ far-red (Figure 5E) due to an increase in dry matter content of both leaves and stem (Supplementary Figures 5E,F). Neither LUE nor radiation use efficiency (RUE) based on fresh or dry mass were affected by EOP far-red treatments (Table 3).

**TABLE 3:**
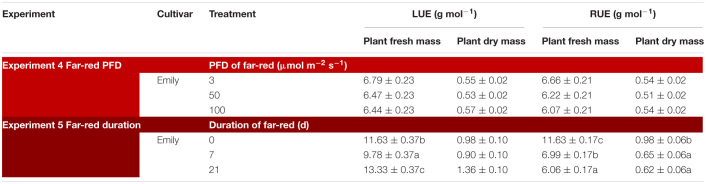
Overview of light use efficiency (LUE) for plant fresh and dry mass in response to far-red (Experiment 4 and 5).

*LUE is based on PPFD incident on the plants accumulated over the initial (i.e., from transplant until start of treatments) and treatment phase, and the radiation use efficiency (RUE), which is based on PFD incident on the plants. Letters indicate significant differences.*

Plants grown with 1 week of added far-red did not show an increase in plant fresh mass while plant fresh mass increased after 3 weeks (Figure 6B). A similar response was found for leaf area (Figure 6C), where a quadratic response to duration of far-red was found; after 1 week leaf area decreased while it increased after 3 weeks. SLA did not change when far-red was applied on top of 150 μmol m^–2^ s^–1^ red-white light (Figure 6D). The response of LUE based on fresh mass followed the pattern of leaf area (Table 3). No differences were found for LUE based on dry mass whereas RUE based on dry and fresh mass decreased when far-red was added (Table 3). The dry mass partitioning to the leaves had an overall linear decrease with duration of far-red (Figure 6F).

## Discussion

### Increased PPFD Applied as End-Of-Production Treatment Increases Plant Fresh Mass and Dry Matter Content

The effect of LED light on plant growth has previously been investigated in species such as lettuce (Li and Kubota, 2009), spinach, rocket, microgreens (Colonna et al., 2016), and basil (Carvalho et al., 2016; Jensen et al., 2018; Pennisi et al., 2019). However, the effects of both light spectra and PPFD have been found to be species dependent (Cope and Bugbee, 2013; Colonna et al., 2016), and in lettuce and tomato even cultivar dependent (Ouzounis et al., 2015, 2016; Gomez and Jimenez, 2020). This was also found in the present study where certain responses to PPFD and spectra were shown to be cultivar dependent. While we found that plant dry matter content in two sweet basil cvs., Emily and Dolly (Figure 1) increased linearly with PFFD, a saturation response was found for the fresh mass of leaves (Supplementary Figure 1A) in cv. Dolly whereas in cv. Emily a linear response to the increase in PPFD was observed. This is in line with results from Pennisi et al. (2020) where fresh and dry mass of both lettuce and basil saturated at a light intensity of 250 μmol m^–2^ s^–1^. A light saturation response occurs when plant growth gets limited by other factors, e.g., CO_2_, temperature or nutrients (Osmond, 1983). Under high light, photosynthesis becomes CO_2_ limited and thus the growth is hampered (Long and Bernacchi, 2003). However, the light intensity at which net photosynthesis gets light limited is species dependent and dependent on the growth environment. Basil, grown under increasing light intensities from 160–310 μmol m^–2^ s^–1^ showed a saturating net leaf photosynthesis at above 220 μmol m^–2^ s^–1^, yet shoot fresh mass and dry matter content increased linearly with light intensity (Dou et al., 2018). A high dry matter content, as observed at higher PPFD, implicates higher levels of carbohydrates. In postharvest storage carbohydrates are used for respiration. Therefore, having a large reserve of carbohydrates are beneficial for shelf-life and quality (Dorais et al., 2002; Caleb et al., 2016). This has also been shown in lettuce (Woltering and Witkowska, 2016) and broccoli (Finger et al., 1999). Consequently, basil with a higher dry matter content might have a better postharvest quality.

Optimal PPFD for basil growth (i.e., highest LUE for plant dry mass) has been suggested to be 250 μmol m^–2^ s^–1^ (DLI 14.4 mol m^–2^ d^–1^) (Pennisi et al., 2020), 224 μmol m^–2^ s^–1^ (DLI 12.9 mol m^–2^ d^–1^) (Dou et al., 2018) and 500 μmol m^–2^ s^–1^ (DLI 28.8 mol m^–2^ d^–1^) (Beaman et al., 2009). In our study, the LUE based on dry mass was the highest at 600 μmol m^–2^ s^–1^ for both cv. Emily and cv. Dolly. For growers the LUE based on fresh mass is probably more interesting, however, at 600 μmol m^–2^ s^–1^ LUE based on fresh mass was the lowest for both cultivars (Table 2). Furthermore, at 600 μmol m^–2^ s^–1^ the leaves were brittle and broke easily; this high light level can therefore not be considered optimal. The combination of initially raising the plants at a PPFD of 150 μmol m^–2^ s^–1^ and an EOP of 300 μmol m^–2^ s^–1^ resulted in an increase in dry mass partitioning to the leaves for both cvs. Emily and Dolly. Whereas an initial PPFD >150 μmol m^–2^ s^–1^ for cv. Dolly resulted in a slightly higher dry mass partitioning to the stem. Therefore, we overall consider the combination of initially raising the plants at a PPFD of 150 μmol m^–2^ s^–1^ and an EOP of 300 μmol m^–2^ s^–1^ (DLI 19.4 mol m^–2^ d^–1^) the optimal growth conditions. This consideration, is based on both LUE parameters and growth and morphological parameters (e.g., dry matter content and leaf area).

### Plant Biomass Does Not Respond to the Fraction of Blue Light in the Spectrum

Blue light and red:blue ratio have been intensely studied. Red light is the most efficient color driving photosynthesis but adding blue to a red background improves the overall photosynthesis (Hogewoning et al., 2010). Yet, the optimal fraction of blue light to a growth light spectrum has had varying conclusions. A blue light optimum was found of 12% blue, in tomato, with respect to leaf dry mass (Kaiser et al., 2019). In basil, Pennisi et al. (2019) found that an increase in blue light up to 58% blue reduced plant fresh mass. However, opposite results were found where an increase in blue fraction up to 37% increased plant fresh mass Piovene et al. (2015) and Jensen et al. (2018) found that a fraction of blue of 60% increased the leaf dry matter content. In our experiments, the fraction of blue mostly affected plant height in cv. Dolly as well as in the purple cv. Rosie. In line with this finding, stem fresh mass and dry mass increased at higher fraction of blue. This may have happened at the expense of leaf fresh mass and dry mass (Supplementary Figure 2). There were no significant effects of fraction of blue light (range 9–100%) on overall plant fresh mass (Figures 2B, 3B, 4B) nor on plant dry mass (Supplementary Figures 2–4) or plant dry matter content (Figures 2E, 3E). Previous findings by Snowden et al. (2016) indicated that an interaction between light intensity and fraction of blue light existed. In the current experiment we found a limited interaction of blue light and light intensity, i.e., only on the dry mass of the leaves (Supplementary Figure 4C) in the green cv. Dolly. For Experiment 3 the purple cv. Rosie was chosen a long with the green cv. Dolly and as in line with results from Dou et al. (2019) the purple cultivar had a lower plant fresh and dry mass of leaves than the green cultivar (Figures 3B, 4B and Supplementary Figures 3C, 4C). However, Dou et al. (2019) found a negative effect of blue light in the purple cultivar and not in green basil whereas blue light did not affect biomass in cv. Rosie in Experiment 3.

Based on our findings a spectrum with 9% blue while the remaining part of the spectrum being 70% red and 19% green can be maintained throughout the growth of basil as an increased amount of blue did not improve the plant fresh mass nor the dry matter content. Furthermore, no differences were found between the EOP and throughout the growth blue light treatment.

### A High Fraction of Blue Light Induces SAS Like Responses

Amongst morphological parameters plant height is one that has been recorded in numerous studies. Blue light usually suppresses elongation (Laskowski and Briggs, 1989) but in a number of cases a promotion of stem elongation has been observed (Johnson et al., 2020) depending on the species and fraction of blue in the spectra (90–100%) (Kong et al., 2018). Blue light is sensed by photoreceptors such as cryptochromes, phototropins, and Zeitlupes (Huché-Thélier et al., 2016). However, phytochromes also absorb blue and consequently blue light can affect the PSS value which indicates the active phytochromes out of the total phytochromes (Sager et al., 1988; Casal, 2013; Meng et al., 2019). A low PSS value results in shade avoidance syndrome (SAS). Hundred percent blue light has been found to increase stem elongation due to low phytochrome activity (PSS 0.49) (Kong et al., 2018; Johnson et al., 2020). In accordance with Kong et al. (2018), we also observed increased stem elongation under both 90 and 100% blue light (Figures 2A, 3A, 4A). This was likely because of the reduced PSS values of 0.7 at 90% blue and 0.49 at 100% blue. Furthermore, the response to blue light on plant height was more pronounced under a low light intensity compared to the high light intensity in cv. Dolly. This finding was also reported by Johnson et al. (2020) although the response was found to be species dependent. In addition, SAS can lead to an increase in leaf area (Demotes-Mainard et al., 2016) but the response is not universal but rather species dependent. Although, 100 and 90% blue induced stem elongation, we found 100% blue to reduce leaf area while 90% blue had no effect. In addition, leaf area decreased overall with increasing fraction of blue light (Figure 2C) which is in accordance with Kaiser et al. (2019).

### Far-Red Increases Plant Height While Effects on Biomass Depend on Duration of Far-Red Application

Shade avoidance syndrome like responses were also found when we grew plants under additional far-red light (Figures 5A, 6A) where plant height increased with far-red intensity and duration. However, leaf area, similar as in the experiments with blue light EOP, decreased when far-red was applied EOP for 5 days or 1 week (Figures 5C, 6C). Increased leaf area in response to far-red has been found to be more pronounced in the early growth stage (Kalaitzoglou et al., 2019) which is in agreement with the increase in leaf area when far-red was added throughout the growth period (Figure 6C). SLA decreased with increasing far-red PFD during the 5-days EOP treatment due the decrease in leaf area and no difference in the dry mass of leaves (Supplementary Figure 5C). SLA which is an indicator of the leaf thickness and would be expected to increase with increasing far-red and decreasing PSS values as found in other species (Ji et al., 2019; Zou et al., 2019). Interestingly, cv. Emily grown with a PSS of 0.62 did not have any change in SLA (Figure 6D). Therefore, we suggest that SAS response in basil is mostly linked to stem elongation. While plant dry matter content increased quadratically under 5 days of EOP far-red (Figure 5E) no increase was found in the longer duration far-red (Figure 6E). However, for the plant fresh mass the longer duration of far-red had a significant effect, mainly due to an increase in stem fresh mass (Supplementary Figure 6B) which also resulted in a lower dry mass partitioning to the leaves. This is in accordance with previous findings, where stem dry mass increased with far-red (Ji et al., 2019).

Recently, Zhen and Bugbee (2020) suggested far-red photons to be photosynthetically active. They found the magnitude of the increase in net photosynthesis to be species dependent where basil was one of the less responsive species. An increase in net photosynthesis is expected to be reflected in an increase in biomass. This was not the case in either of our experiments as cv. Emily did not increase in plant dry mass after 5 days (total PFD 350–400 μmol m^–2^ s^–1)^ (Supplementary Figure 5) or after 1 week of added far-red (total PFD 330 μmol m^–2^ s^–1^) (Supplementary Figure 6). However, when the PPFD increased from 150 to 300 μmol m^–2^ s^–1^ dry mass did increase (Supplementary Figure 1). The decrease in fresh mass after 1 week of added far-red and subsequent increase after 3 weeks could indicate an acclimation period (Figure 6B). This is supported by our results with increased far-red intensity where no increase in plant fresh mass was found (Figure 5B). Although, the radiation use efficiency for plant fresh mass increased by 3 weeks of added far-red (Table 3) it decreased based on dry mass with increasing duration of far-red in Experiment 5 while far-red did not affect radiation use efficiency in Experiment 4. Thus, additional far-red, in a small dosages added 5 days before harvest may be beneficial to improve leaf dry matter content whereas a higher dosage throughout the growth does not yield a desired plant morphology as the stem is not a used organ from basil plants.

## Conclusion

We showed that growth (plant fresh mass, plant dry matter content, and dry mass partitioning to the leaves) and morphology (plant height and leaf area) were significantly affected by EOP increase in PPFD. Interestingly, LUE based on fresh mass decreased with increasing PPFD whereas LUE based on dry mass increased. The plant fresh mass did not respond to the fraction of blue light while plant dry matter content was reduced at the combination high fraction of blue and a high PPFD. When the spectrum consisted of either 90 or 100% blue, either applied as EOP treatments or throughout the growth shade avoidance syndrome was induced and plants grew taller resulting in more fresh and dry mass partitioned to the stem. Therefore, a high fraction of blue in the spectrum is not desirable for basil growth as the leaves are the consumed part.

Addition of far-red for basil during growth is most beneficial when added as EOP treatment before harvest and only in a lower dosage at a high PPFD as it increases dry matter content of both leaves and stem.

## Data Availability Statement

The raw data supporting the conclusions of this article will be made available by the authors, without undue reservation.

## Author Contributions

DL, EW, CN, and LM conceptualized the research plan. DL, EW, and LM designed the experiments. DL carried out the experiments, analyzed the data, and wrote the manuscript. EW and LM provided critical feedback on the manuscript. CN provided critical comments to the overall structure of the manuscript. All authors reviewed and approved the final manuscript.

## Conflict of Interest

CS Nicole was employed by company Signify. The remaining authors declare that the research was conducted in the absence of any commercial or financial relationships that could be construed as a potential conflict of interest.
