# Supplementary material for: Response of Basil Growth and Morphology to Light Intensity and Spectrum in a Vertical Farm
Source: Front Plant Sci. 2020 Dec 4;11:597906. doi: 10.3389/fpls.2020.597906 (PMC7793858; doi:10.3389/fpls.2020.597906)
Supplement: Supplementary file 1 [file Table_1.docx]

Supplementary Material

## Supplementary Figures


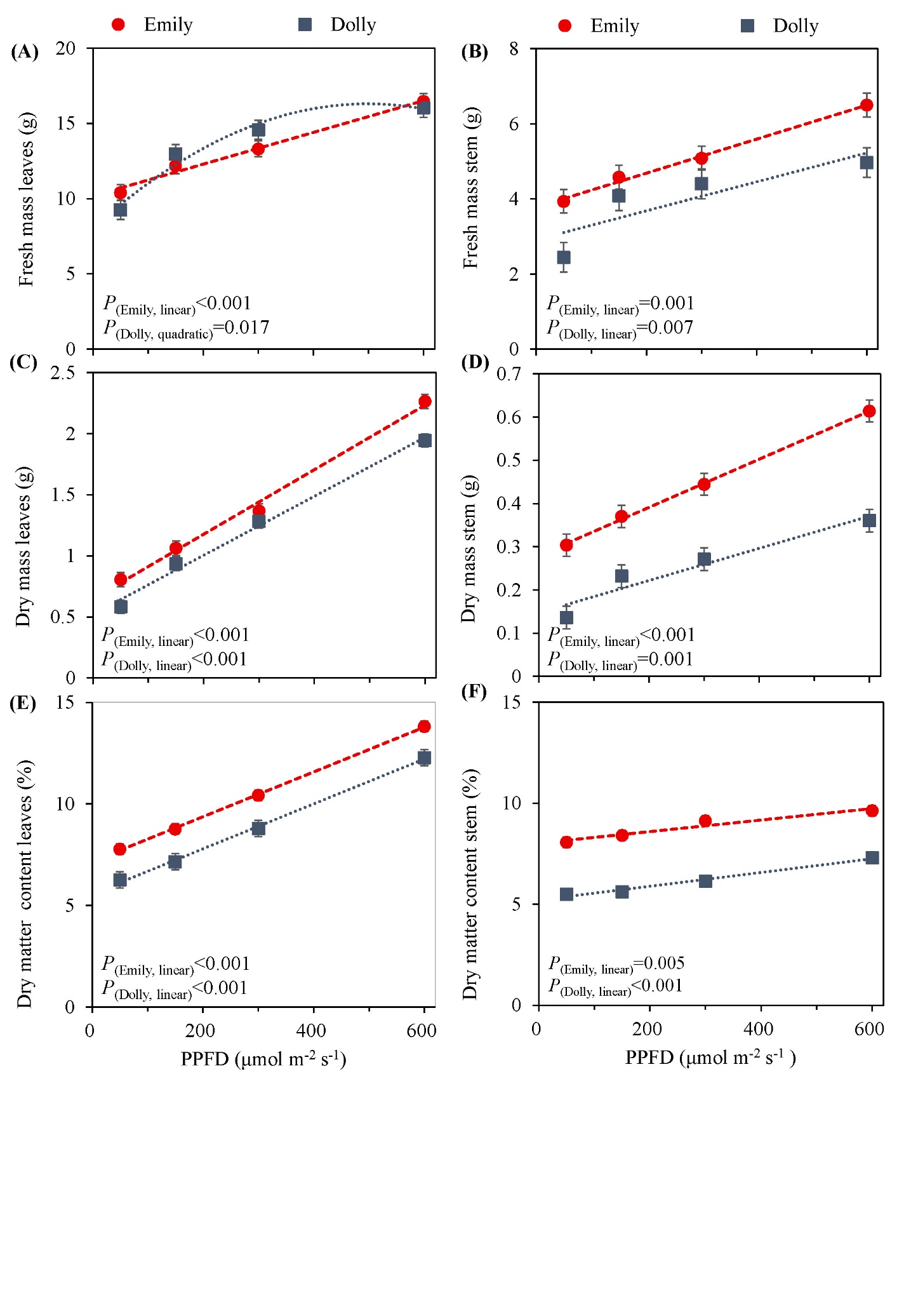


**Figure S1**. Response of basil cvs. Emily (red circles) and Dolly (grey squares) to different End-Of-Production PPFD. Plants were grown for 30 days under 150 µmol m^-2^ s^-1^ after which they were exposed to different PPFD (i.e. 50, 150, 300 and 600 µmol m^-2^ s^-1^) during 5 days before harvest. **(A)** Fresh mass leaves, **(B)** fresh mass stem, **(C)** dry mass leaves **(D)** dry mass stem, **(E)** dry matter content leaves, **(F)** dry matter content stem. Data are means of 3 blocks (n=3) each with six replicate plants. Error bars represent standard errors of means, when larger than symbols. For significant quadratic or linear effects of PPFD, trendlines together with the respective *p*-values (α=0.05) are depicted.

**
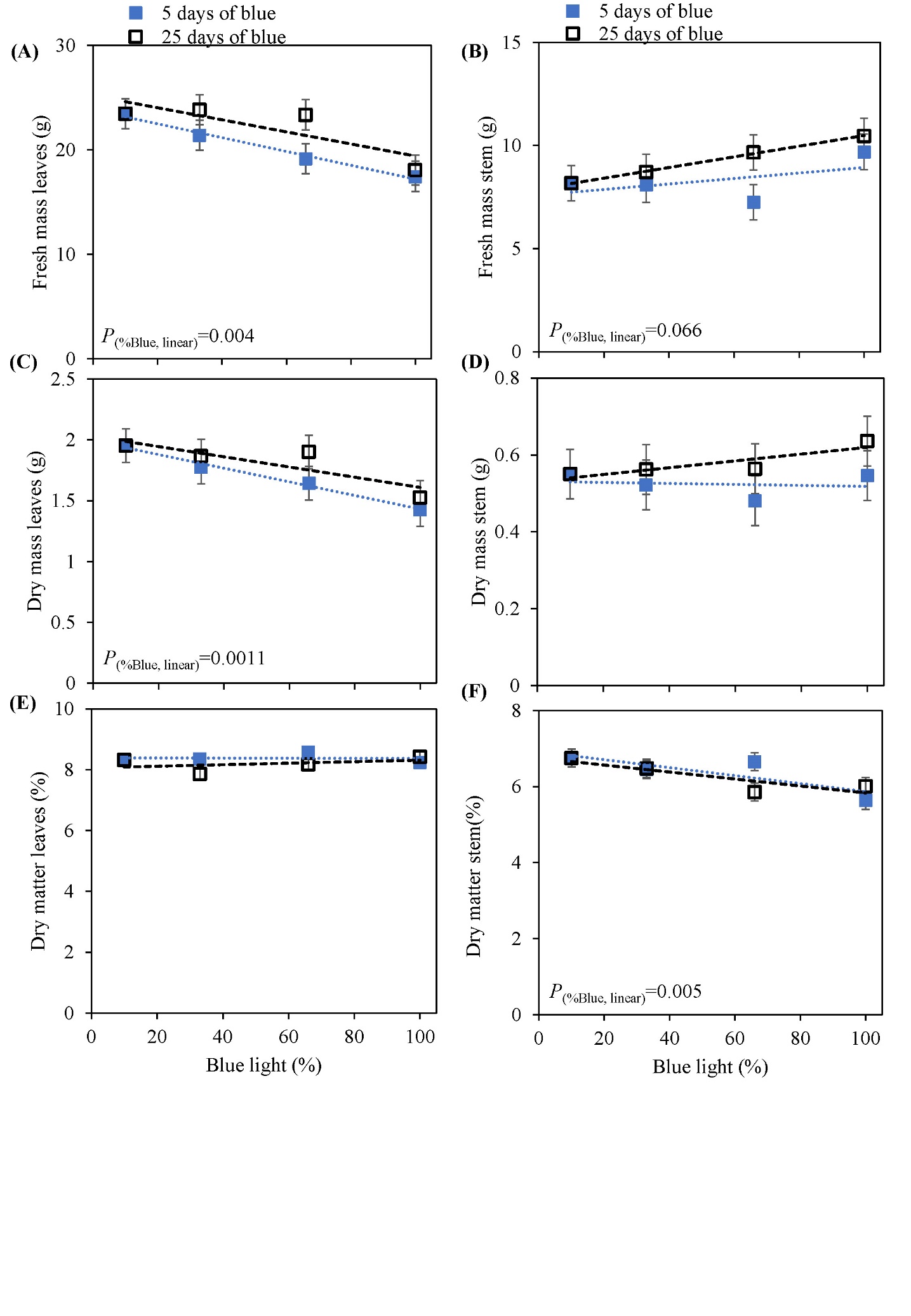
Figure S2**. Response of basil cv. Dolly to different blue fractions out of a total PPFD of 300 µmol m^-2^ s^-1^ either applied throughout-the-growth for 25 days (open squares) or as 5 days End-Of-Production treatments (closed squares). The data point 9% blue is shared between 5 days and 25 days as 9% blue light also was the initial phase before EOP treatments. **(A)** Fresh mass leaves, **(B)** fresh mass stem, **(C)** dry mass leaves **(D)** dry mass stem, **(E)** dry matter content leaves, **(F)** dry matter content stem. Data are means of 2 blocks (n=2) each with six replicate plants. Error bars representing standard errors, when larger than symbol size. For significant quadratic or linear effects of increasing fraction of blue, trendlines together with the respective *p*-values (α=0.10) are depicted.

**
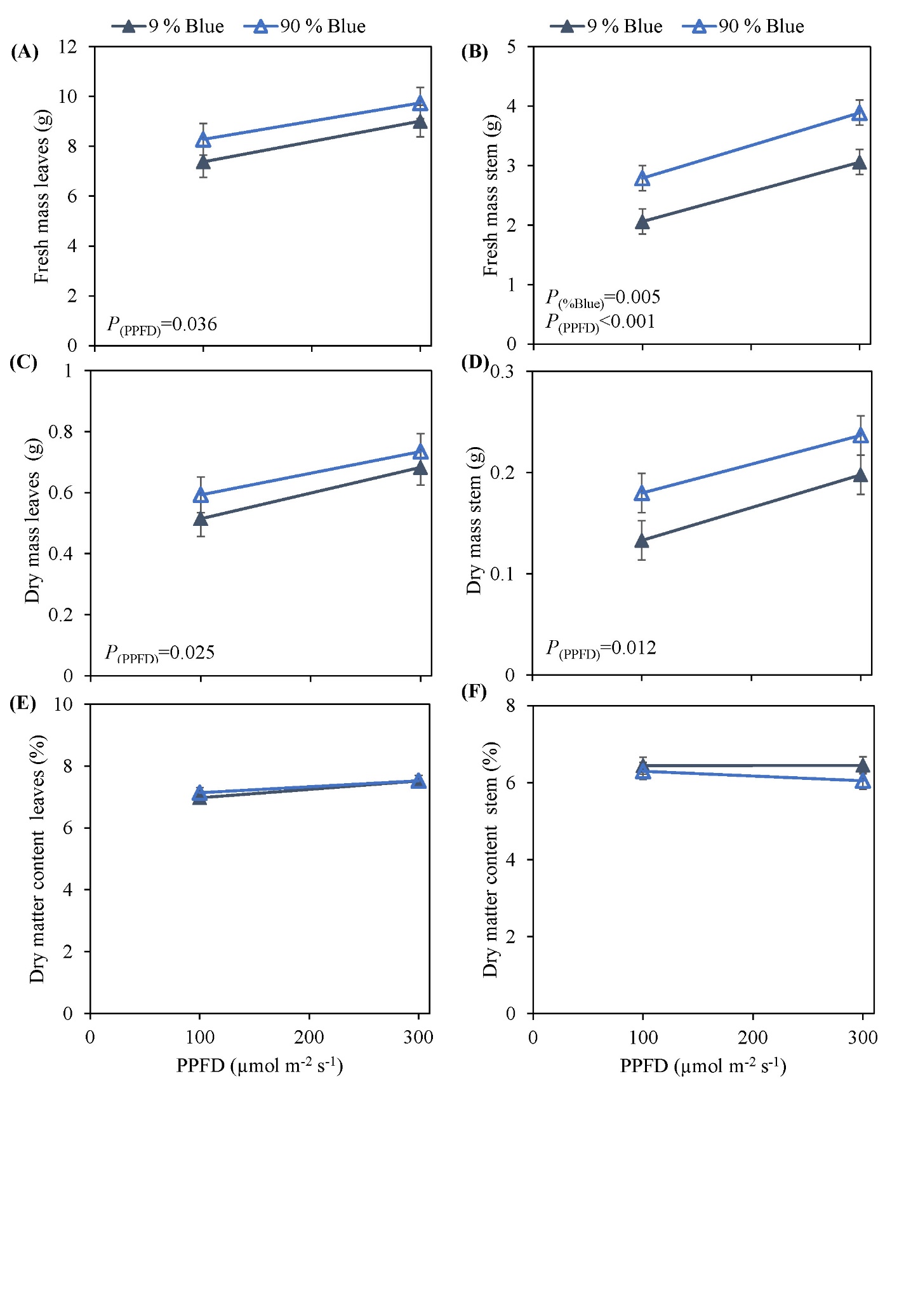
Figure S3.** Response of basil cv. Rosie to End-Of-Production blue light and PPFD. Plants were grown for 30 days under red white light (9% blue) and PPFD of 200 µmol m^-2^ s^-1^. EOP treatments were applied five days before harvest blue light and PPFD were changed to 100 µmol m^-2^ s^-1^ red white with 9 % blue or 90 % blue, and to 300 µmol m^-2^ s^-1^ red white with 9 % blue or 90 % blue. Closed triangle 9% blue and open triangle 90% blue. **(A)** Fresh mass leaves, **(B)** fresh mass stem, **(C)** dry mass leaves **(D)** dry mass stem, **(E)** dry matter content leaves, **(F)** dry matter content stem. Data are means of 4 blocks (n=4) each with six replicate plants. Error bars representing standard errors, when larger than symbol size. *P*-values of main effects %Blue and PPFD (α=0.05) are depicted.


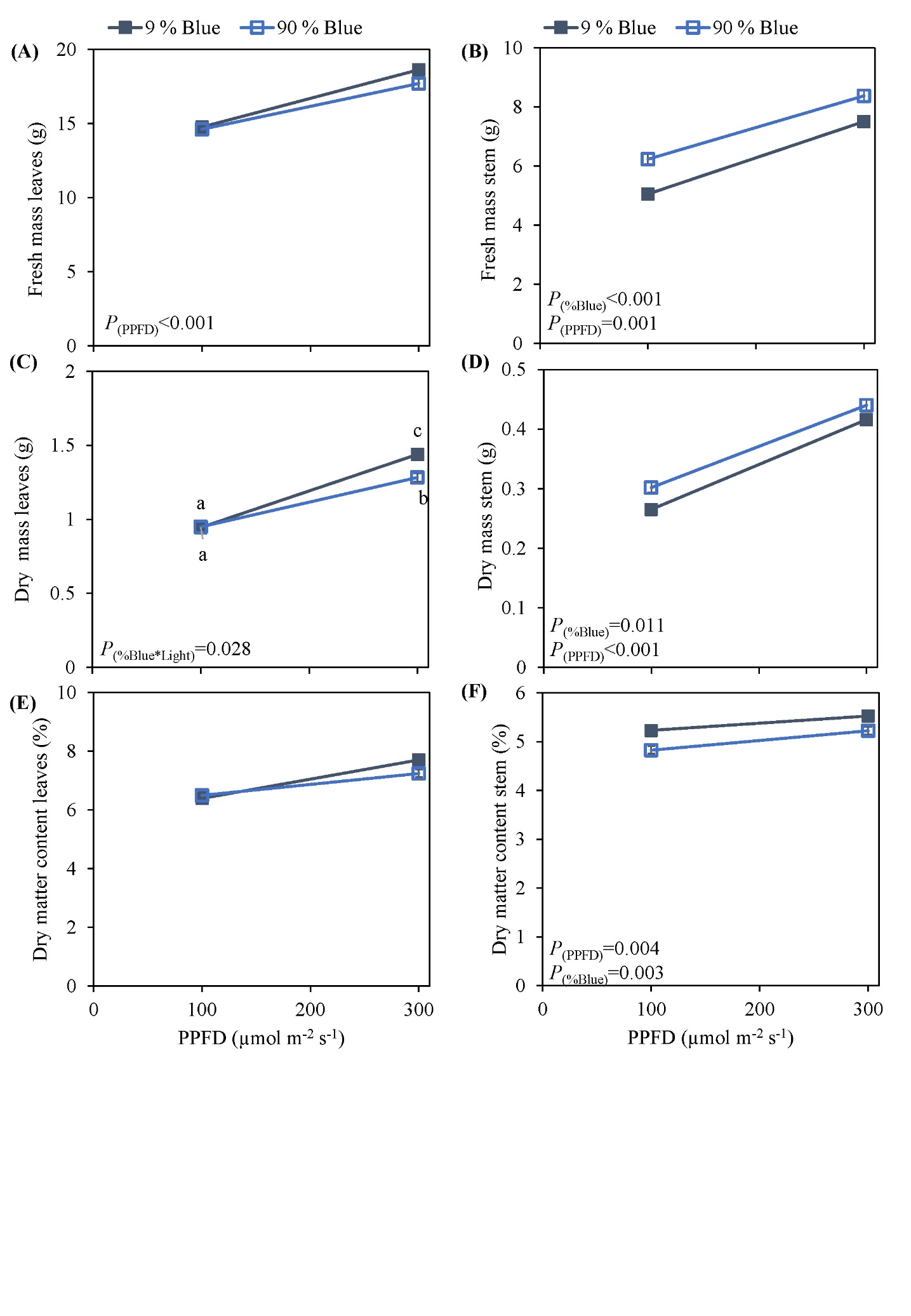


**Figure S4.** Response of basil cv. Dolly to End-Of-Production blue light and PPFD. Plants were grown for 30 days under red white light (9% blue) and PPFD of 200 µmol m^-2^ s^-1^. EOP treatments were applied five days before harvest blue light and PPFD were changed to 100 µmol m^-2^ s^-1^ red white with 9 % blue or 90 % blue, and to 300 µmol m^-2^ s^-1^ red white with 9 % blue or 90 % blue. Closed squares 9% blue and open squares 90% blue. **(A)** Fresh mass leaves, **(B)** fresh mass stem, **(C)** dry mass leaves **(D)** dry mass stem, **(E)** dry matter content leaves, **(F)** dry matter content stem. Data are means of 4 blocks (n=3) each with six replicate plants. Error bars representing standard errors, when larger than symbol size. *P*-values of main effects %Blue and PPFD (α=0.05) are depicted.


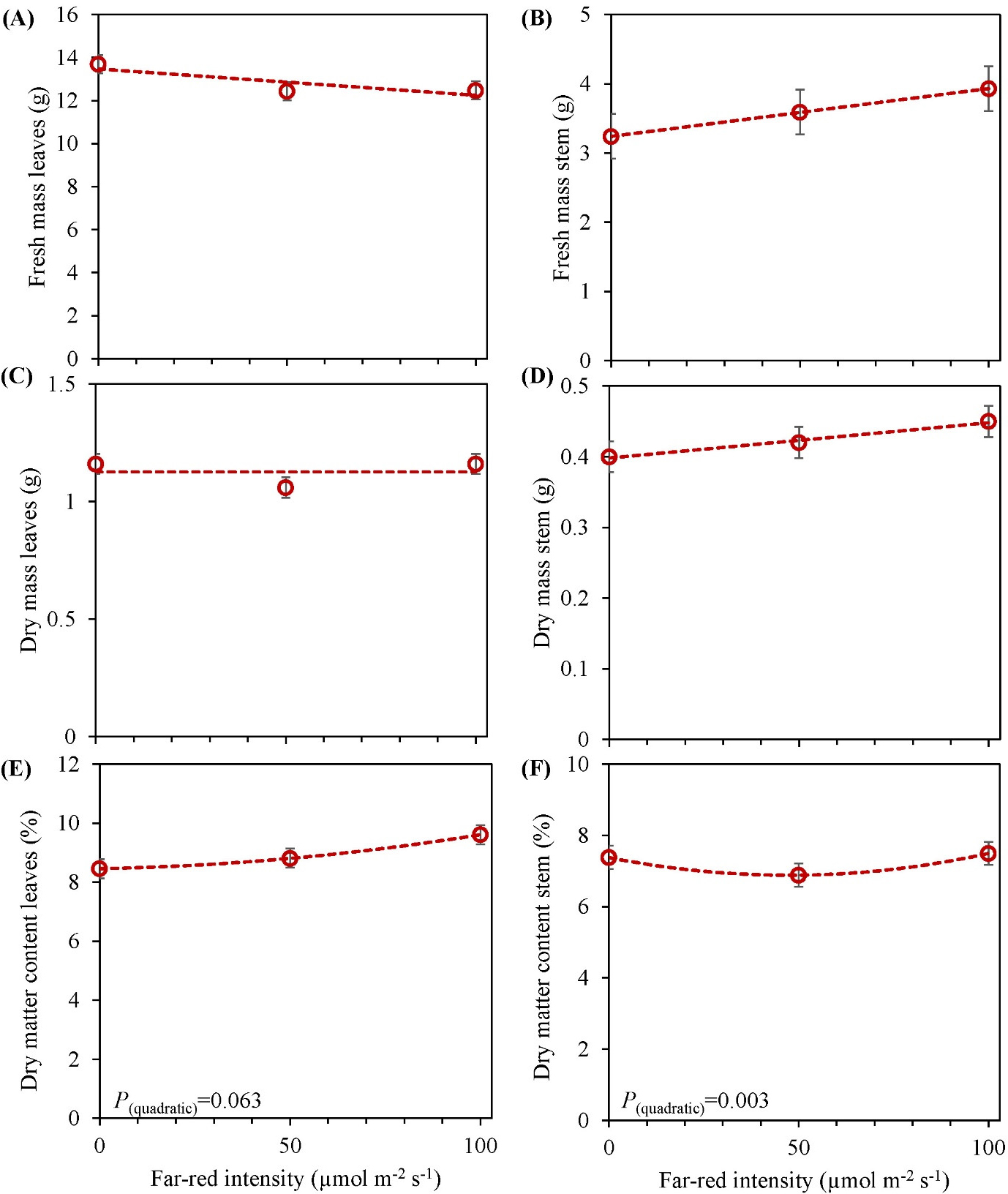


**Figure S5.** Response of basil cv. Emily to End-Of-Production increased far-red PFD. Plants were grown for 15 days under PPFD 150 µmol m^-2^ s^-1^, after transplant for another 15 days of PPFD 300 µmol m^-2^ s^-1^ red white light and exposed to different far-red intensities (i.e. 0, 50, 100 µmol m^-2^ s^-1^ in addition to 300 µmol m^-2^ s^-1^ red white light applied during 5 days before harvest. **(A)** Fresh mass leaves, **(B)** fresh mass stem, **(C)** dry mass leaves **(D)** dry mass stem, **(E)** dry matter content leaves, **(F)** dry matter content stem. Data are means of 2 blocks (n=2) each with five replicate plants. Error bars representing standard errors, when larger than symbol size. For significant quadratic or linear effects of increasing far-red intensity, trendlines together with the respective *p*-values (α=0.10) are depicted.


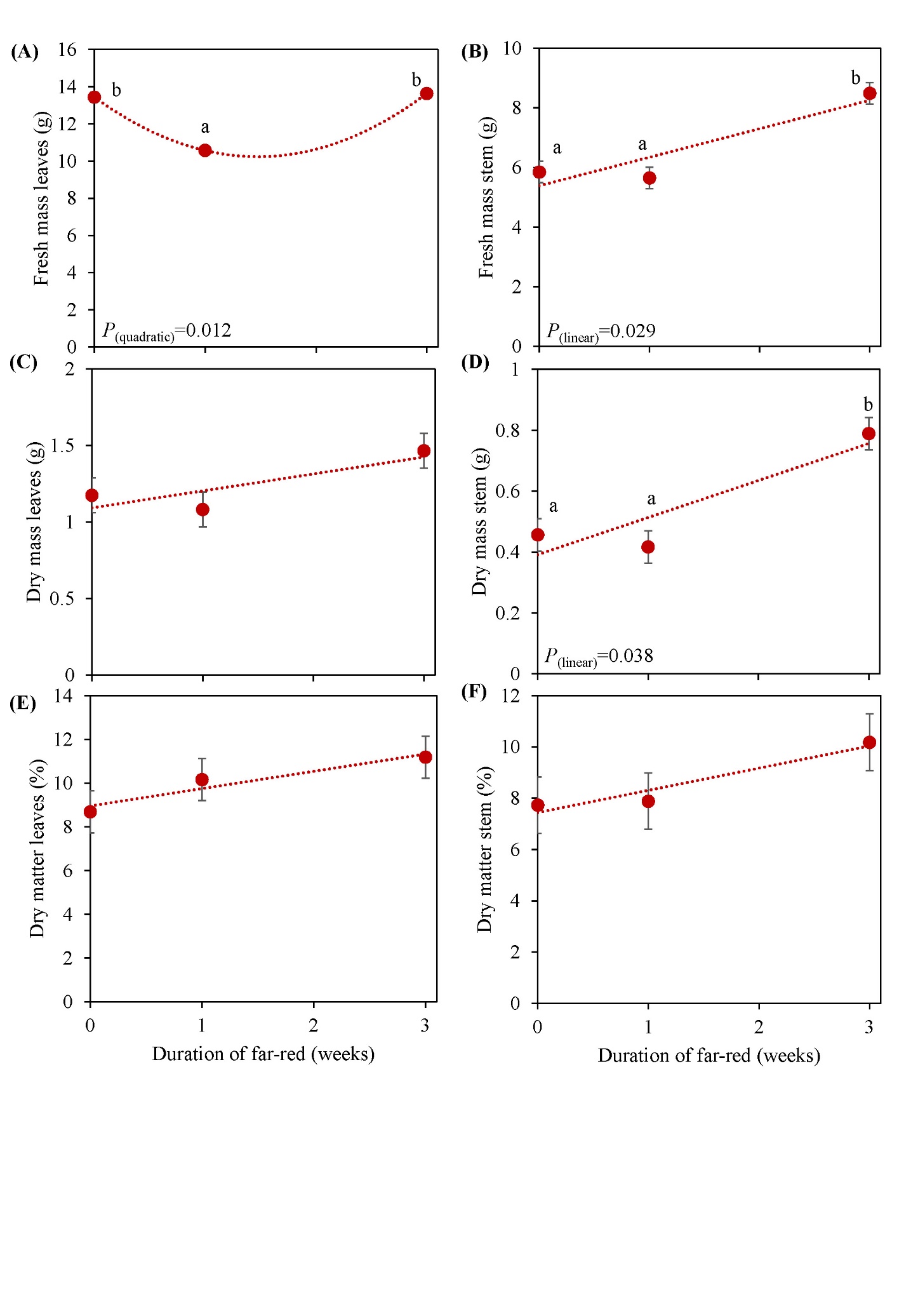


**Figure S6.** Response of basil cv. Emily to different duration of far-red treatments either throughout-the-growth for three weeks or as one week End-Of-Production treatment. Plants were grown for 31 days under 150 µmol m^-2^ s^-1^ red white light, and additional far-red light (180 µmol m^-2^ s^-1^) was applied during 0, 1 and 3 weeks before harvest.. **(A)** Fresh mass leaves, **(B)** fresh mass stem, **(C)** dry mass leaves **(D)** dry mass stem, **(E)** dry matter content leaves, **(F)** dry matter content stem. Data are means of 2 blocks (n=2) each with five replicate plants. Error bars representing standard errors, when larger than symbol size. For significant quadratic or linear effects of duration of far-red, trendlines together with the respective *p*-values (α=0.10) are depicted.
